# A multiplex method for high-throughput quantification of conjugated saccharide in glycoconjugate vaccines

**DOI:** 10.1007/s00216-025-06249-5

**Published:** 2025-11-29

**Authors:** Giovanni Santostefano, Alessio Corrado, Carmine Malzone, Leonardo Mori, Diego Amendola, Stefania Berti, Valeria Di Bussolo, Riccardo De Ricco

**Affiliations:** 1 University of Pisa, Pharmacy Department, Via Bonanno Pisano 33, 56126 Pisa, Italy; 2 Technical R&D, GSK, Via Fiorentina 1, 53100 Siena, Italy

**Keywords:** Glycoconjugate vaccine, Conjugated saccharide, Critical quality attributes, Bio-analytical assays, Luminex, Multiplex

## Abstract

Glycoconjugate vaccines rely on the accurate quantification of its active ingredient, also called conjugated saccharides (CS), which is usually estimated from the indirect measurement of total and free (unconjugated) saccharide to ensure product quality and efficacy. Conventional methods like HPAEC-PAD often require harsh and time-consuming sample pre-treatment, especially in multivalent formulations. In this study, we introduce a tetraplex sandwich immunoassay based on Luminex technology, capable of directly and simultaneously quantifying the conjugate saccharide in MenACWY vaccine without pre-treatments (e.g., hydrolysis, solid-phase extraction, and ultrafiltration steps). The new assay exhibits robust specificity, sensitivity, linearity, precision, and accuracy across a broad concentration range, and effectively discriminates conjugated saccharides from free saccharides and carrier protein. Stability studies under both accelerated and long-term conditions confirm the method’s suitability as a stability-indicating assay. This high-throughput, multiplex platform offers a robust and efficient alternative to traditional physico-chemical approaches, streamlining analytical workflows for increasingly complex glycoconjugate vaccines.

**Graphical Abstract:**

**Figure:**
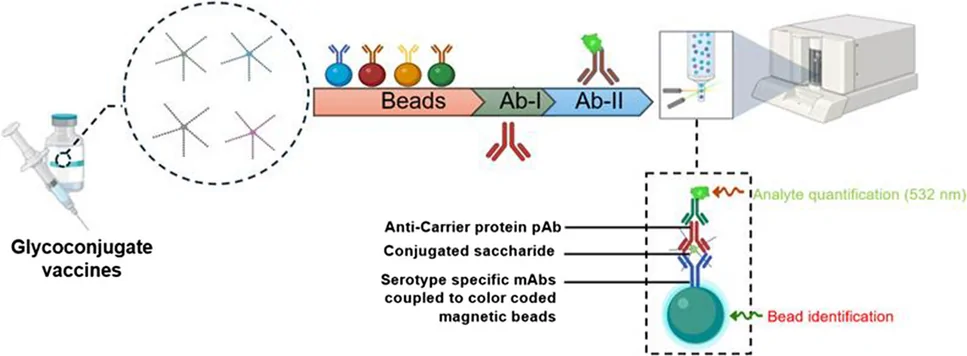

**Supplementary Information:**

The online version contains supplementary material available at https://doi.org/10.1007/s00216-025-06249-5.

## Introduction

Over the past decades, vaccines have emerged as one of the most effective and economically viable public health interventions, profoundly impacting global health by drastically reducing the burden of infectious diseases [1, 2]. Their pivotal role in controlling—and in some cases eradicating—deadly viral and bacterial infections underscores their transformative value. Within this context, glycoconjugate vaccines represent a significant advancement in vaccinology, particularly for their ability to induce protective immunity in populations that traditionally show limited responsiveness to plain polysaccharide vaccines, such as infants and young children [3]. Glycoconjugate vaccines operate through the covalent linkage of bacterial polysaccharides to a carrier protein, converting T cell-independent antigens into T cell-dependent responses, facilitating the establishment of immunological memory and enhancing antibody persistence [4]. Such vaccines have proven especially effective in inducing robust and durable immune responses against encapsulated pathogens including *Haemophilus influenzae type b* (Hib), *Streptococcus pneumoniae*, *Salmonella typhi*,* and Neisseria meningitidis*, with a very limited amount of saccharide injected [5]. The widespread integration of glycoconjugate vaccines into national immunization programs reflects their critical role in infectious disease prevention [6]. However, it also highlights the necessity for rigorous analytical control strategies to ensure vaccine quality, safety, and effectiveness. Precise characterization of key vaccine attributes is essential for product consistency and regulatory compliance [7]. Among these attributes, it is fundamental to fully characterize the content of conjugated saccharide (CS), the active pharmaceutical ingredient, and free saccharide (FS), a process- and stability-related impurity. Over the years, extensive technical research has focused on the analytical development of glycoconjugates against *Neisseria meningitidis* (serotypes A, C, W, Y, X) [8], particularly employing high-performance anion exchange chromatography with pulsed amperometric detection (HPAEC-PAD) [9–12]. HPAE-PAD is recognized as the principal physico-chemical method for saccharide analysis, followed by colorimetric methods. However, the employment of these techniques on the final drug product (DP) containing *Neisseria meningitidis* antigens poses challenges. These are mainly due to the presence of some saccharide repeating units in multiple polysaccharides (e.g., MenW and MenY), resulting, upon hydrolysis, in overlapping peaks. This effect can be induced also by the matrix (e.g., sucrose or lactose), requiring specific and lengthy pre-treatment for its removal [13, 14]. Additionally, traditional HPAEC-PAD methods proved to be time-consuming and material-intensive due to the necessity of conducting two separate assays—one for determining the total saccharide (TS, conjugated + free) and another for the free saccharide—to estimate the CS content [15]. Recently, Rech et al. [16] developed a new HPAEC-PAD method that directly quantifies the conjugated saccharide of MenA-CRM_197_ in Menveo, replacing the traditional indirect approach (CS = TS − FS). This has been an innovative approach toward the quantification of glycoconjugate vaccines: the active ingredient is directly measured, providing a more precise and reliable measurement of the antigen. This new method also aims to enhance high-throughput capabilities of that technology. The direct estimation of the conjugated saccharide proved to be effective for quality control of the final multivalent vaccine and has been favorably accepted by regulatory authorities. However, this technique still requires sample pre-treatment and customized hydrolysis. To address these limitations, particularly in multivalent contexts, alternative technologies have been explored. Among the emerging solutions, Luminex-based assays have demonstrated significant potential. xMAP technology represents an evolution of traditional enzyme-linked immunoassay (ELISA), with the additional characteristic of the multiplex approach that enables the simultaneous detection and quantification of multiple biomolecules within a single assay. This is possible using color-coded microspheres, each with a unique color signature, which can be coated with specific capture molecules such as antibodies or antigens. This allows the measurement of multiple targets concurrently, as different sets of beads are incorporated into one sample to capture various analytes. After adding the beads to the sample, the target molecules bind to their corresponding coated beads, and a detection antibody with a fluorescent dye, generally phycoerythrin (PE), further binds to these targets. Then the instrument utilizes lasers to identify each bead by its specific color and quantify the fluorescence intensity from the detection dye, which correlates to the amount of target molecules bound to the bead [17, 18]. Therefore, considering the potential of Luminex xMAP technology in multiplexing various antigens in a single well, we applied it on the quadrivalent Menveo lyophilized vaccine as a key study. This paper describes the development and feasibility of using Luminex xMAP technology as an innovative application for simultaneously analyzing and quantifying all antigens in a single high-throughput assay, without any pre-treatment for separating free and conjugated saccharide. This approach overcomes the limitations of traditional physico-chemical methods for the MenACWY-CRM_197_ conjugate vaccine, significantly enhancing efficiency and streamlining the analytical process for consistently increasing valency in vaccine formulations (e.g., MenACYWX [19] or MenACWY reconstituted with MenB [20]).

## Material and methods

### Beads, antibodies, chemicals, and consumables

Luminex MagPlex-C Microspheres (MC10020-01; MC10025-01; MC10033-01; MC10077-01) are manufactured by Diasorin (Saluggia, Italy). For coupling were used 1-Ethyl-3-(3-dimethylaminopropyl) carbodiimide (cat. 22980) and N-hydroxy-sulfosuccinimide (cat. 24510) chemical agents, both from ThermoFisher (Waltham, MA, USA). For buffer preparation PBS tablets (cat. 09–8912-100) from Medicago (Quebec City, Canada), Tween20 (cat. P1379) and BSA (cat. A3983) from Sigma Aldrich (Burlington, MA, USA). For the assay were used Greiner plates (cat. 655904) from Sigma Aldrich.

### Buffers

For coupling, sodium phosphate 100 mM (pH = 6.2) for beads activation, MES 50 mM (pH = 5.0) for beads-mAb reaction, PBS 1X, 0.5% BSA, 0.05% Tween20 for beads saturation; PBS 1X, 0.05% Tween20 for beads washing and storage. During the assay, NaCl 9.0 g/L were used for vaccine reconstitution or for preparing drug substances mixtures, PBS 1X, 0.05% Tween20 for sample/beads dilution and plate washing, and PBS 1X used for plate reading.

### Antibodies

Serotype-specific mouse monoclonal antibodies (mAb anti-MenA, batch 28022017; mAb anti-MenC, batch 13072017; mAb anti-MenW, batch 04052017; mAb anti-MenY, batch 23082017) for beads coupling were provided by the Bioassay department of GSK and generated by Takis (Rome, Italy). Rabbit Anti-CRM_197_ whole antiserum containing pAbs (cat. ab53828) produced by Abcam (Cambridge, Eng) is employed as primary antibodies after previous purification with Ab spin-trap Protein-G columns (cat. 28408347) with appropriate buffer kit (cat. 28903059) from Cytiva (Marlborough, MA, USA). After purification from rabbit sera following manufacturer procedure [21], IgG titers were measured by UV spectrometry at 280 nm. Goat Anti-Rabbit specific Fab conjugated to R-Phycoerythrin (cat. 111–117-008) was purchased from Jackson ImmunoResearch (West Grove, PA, USA) and used as secondary antibody.

### Standards and samples

For this study, the GSK manufacturing site produced both mono-valent bulk conjugates and final lyophilized vaccine and MenACWY antigens coupled to CRM_197_ protein (12 μg of MenA-CRM_197_ antigen and 6 μg of saccharide content for the C, W, Y conjugates). The vials were reconstituted in 600 μL of NaCl 9.0 g/L solution, for a resulting target concentration of 20 μg/mL, 10 μg/mL, 10 μg/mL, 10 μg/mL, respectively, for MenACWY-CRM_197_ conjugates.

### Coupling

To this aim, each anti-serotype mAb has been coupled to a certain color-coded carboxylic bead (anti-MenA mAb, anti-MenC mAb, anti-MenW mAb, anti-MenY mAb to B20-B25-B33-B77 respectively). To this end, the carbodiimide coupling protocol for antibodies and proteins from Luminex was utilized [22]. Following the Luminex procedure, 100 µL of MagPlex-C Microspheres, containing 1.25 million beads, was transferred to a protein low-binding 1.5 mL tube and washed with water. Subsequently, 80 µL of a sodium phosphate solution was added to initiate the bead activation. This reaction was carried out by sequentially adding 10 µL of sNHS at a concentration of 50 mg/mL, followed by 10 µL of EDC at the same concentration. Each tube was vortexed and mixed by 360° rotation for 20 min at room temperature, protected from light. Once activated, the beads were coupled with a specific amount of serotype-specific mAb in a total volume of 500 µL of MES-based buffer. The tubes were then vortexed and mixed by 360° rotation for 2 h at room temperature, covered from light. After the coupling reaction, the beads underwent two washing steps using 500 µL of PBS 1X, 0.05% Tween20. Thus, saturation of unreacted bead carboxylic groups was achieved by adding 500 µL of PBS 1X with 0.05% Tween20 and 0.2% BSA, followed by vortexing and mixing by 360° rotation for 30 min at room temperature, protected from light. Finally, the beads were washed twice with 500 µL of PBS 1X containing 0.05% Tween20, and 1000 µL of the same buffer was added for storage, maintaining a final bead concentration of 1.25 million per mL.

### Assay steps

MenACWY-CRM_197_ lyophilized vaccine samples and standard were initially reconstituted with 600 µL of NaCl 9.0 g/L solution for the target antigens concentration. Subsequently diluted to a concentration of 1:1000. Samples and standard curve were dispensed in duplicate in the first column, while the remaining wells of the plate were filled with sample diluent. Then a 2.5-fold serial dilution was performed for titrating sample and standard curve. Next, the beads mix was prepared by diluting each bead-antibody complex into sample diluent at a ratio of 1:10 and dispensed into each well with a volume of 10 µL. The plates were then incubated at room temperature for 1 h with agitation (800 rpm). Following this incubation, the plates were washed three times with sample diluent using a 405-microplate magnetic washer. Next, 100 µL of diluted and purified anti-CRM_197_ pAb was added to each well, and the plates were incubated again under the same conditions. After a second washing step, 50 µL of a 1:200-diluted anti-rabbit Fc-specific secondary antibody was added to each well, followed by a 30-min incubation at room temperature with agitation (800 rpm). The wells were washed, reconstituted in 100 µL of PBS 1X, and after agitation the plate was read by Luminex at high-sensitivity.

### Instruments, data analysis, and statistics

Bio-plex 200 instrument was used for plate reading and related system Bioplex-Manager 6.0 (Bio-plex ProTM, Bio-Rad Laboratories, Inc., Hercules, CA, USA) was used to read the median fluorescence intensity (MFI), then collected by MicrosoftExcel365 (Microsoft Corporation, WA, USA) and analyzed on the online version of Combistat EDQM (Council of Europe, Strasbourg, FR). Parallel line assay (PLA) was used as a statistical model to analyze log_10_ (Dose)-log_10_ (Response) linear curves, formed by at least four points. For each sample, an analysis of variance (ANOVA) was performed to assess the validation criteria: a significant regression (p < 0.05), absence of non-linearity (p > 0.05), and absence of non-parallelism (p > 0.05). After the verification of these conditions, the sample concentration of the test sample versus the standard was calculated along with confidence intervals.

### HPAEC-PAD

Regarding the chromatography part, we followed the procedure already published by Rech et al. [16] for sample pre-treatment and De Ricco et al. [9] for HPAEC-PAD separation conditions.

## Results

**Fig. 1:**
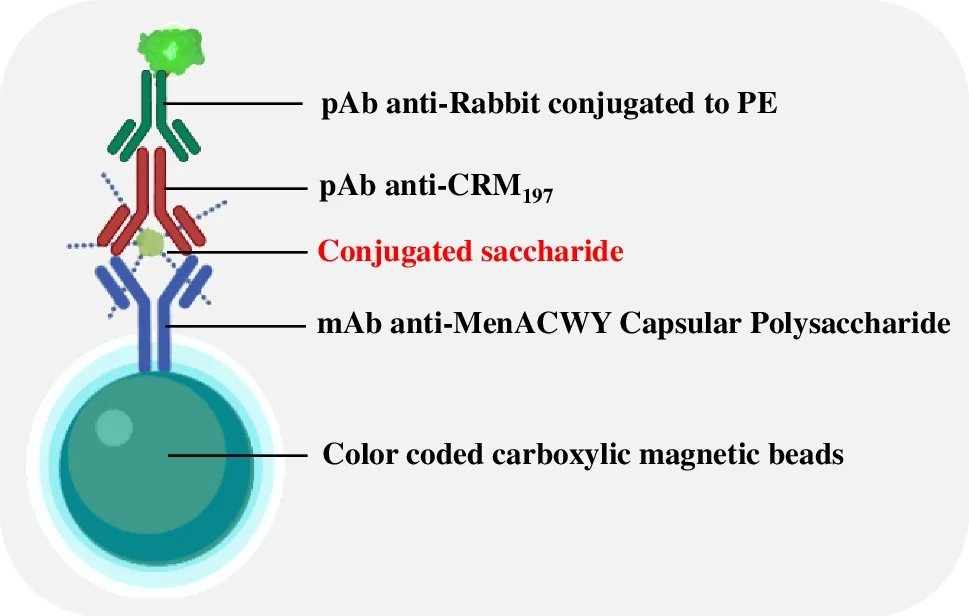
Sandwich configuration against conjugated saccharide in glycoconjugate vaccines. Image created by BioRender.com

To leverage the full potential of Luminex technology and avoid the need for sample pre-treatment, we have designed a configuration that selectively captures and quantifies only the conjugated saccharide of each antigen. This aim can be achieved by coupling each bead with monoclonal antibodies which are specific to MenACWY antigens, serving as serotype-specific capture elements, so that each bead corresponds to a distinct antigen. The conjugate can then be identified using polyclonal antibodies against the carrier protein, i.e., CRM_197_, ensuring specific detection of the conjugate, so well distinct from the free saccharide, as shown in Fig. 1. The subsequent paragraphs will illustrate the development and the results of the challenge that was performed on the method.

### Method development

**Fig. 2:**
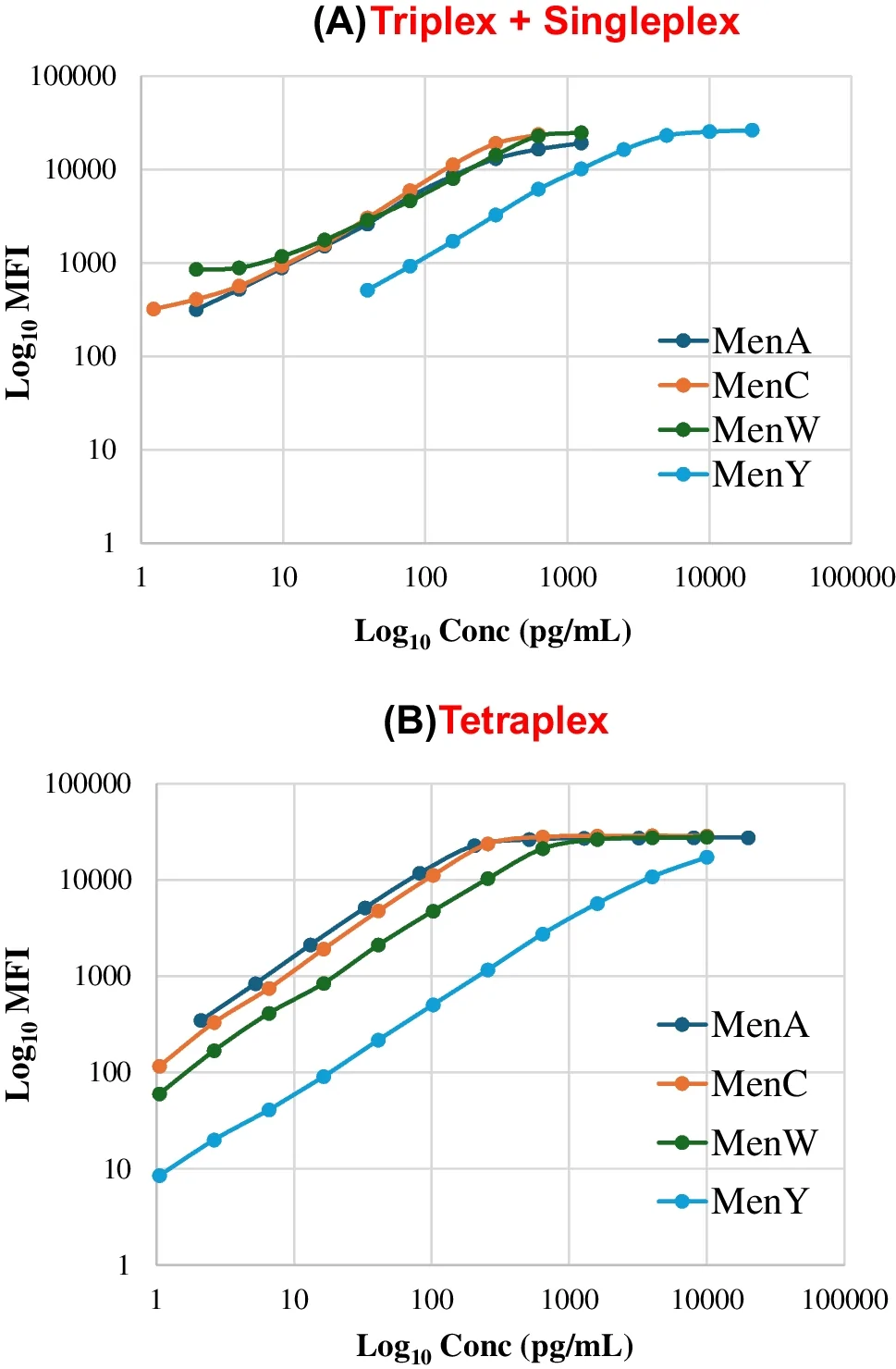
**A** Triplex + singleplex (3 + 1) assay format in which MenACW and MenY conjugates are analyzed separately in two plates with 2 different starting sample dilutions. **B** Tetraplex assay format in which all the antigens are analyzed in the same plate using a common starting dilution and titration step. Picture created on Microsoft Excel365

During coupling, the major parameter that necessitated optimization was the amount of mAb coupled on the beads. Therefore, for each serotype, the mAb amount was doubled, ranging from 0.5 µg to 16.0 µg of mAb for millions (mln) of beads. After this screening, the optimal amount of coupled mAbs resulted in 4.0, 1.0, 2.0, and 4.0 µg for MenA, MenC, MenW, and MenY, respectively. These values were decided based on the ability to achieve the highest and most consistent MFI, lowest blanks, and ultimately the highest S/N ratio. Subsequently, an evaluation was conducted of the initial sample concentration, the titration step, Ab-I, and Ab-II dilution. In this step of the optimization process, both whole IgG-specific and Fc-specific Ab-II have been taken into consideration. The second led to a reduction in unspecific binding, thereby translating into an enhancement of the S/N ratio. Moreover, a great improvement of the latter has been obtained after purification with protein G columns of anti-CRM_197_ whole serum. This purification step has been necessary to improve linearity, LOD/LOQ, and have the lowest blank values, thereby minimizing nonspecific interactions. After setting up all the coupling and assay steps, we optimized the layout to find a common condition for analyzing concomitantly as many antigens as possible. Initially, a triplex + singleplex format was developed (Fig.  2A). For this setup, we used a two-fold dilution step to analyze MenACW and MenY separately across two different plates. However, given the higher resources and time demands of this format, improvements were made to find a unique condition in tetraplex (Fig. 2B) configuration that would allow all four serotypes to achieve a linear regression curve. A longer dilution step and a starting sample concentration were selected, enabling at least five points of calibration, also for MenA, whose linear range is achieved only after further dilution points compared to the other serotypes.

### Specificity

**Fig. 3:**
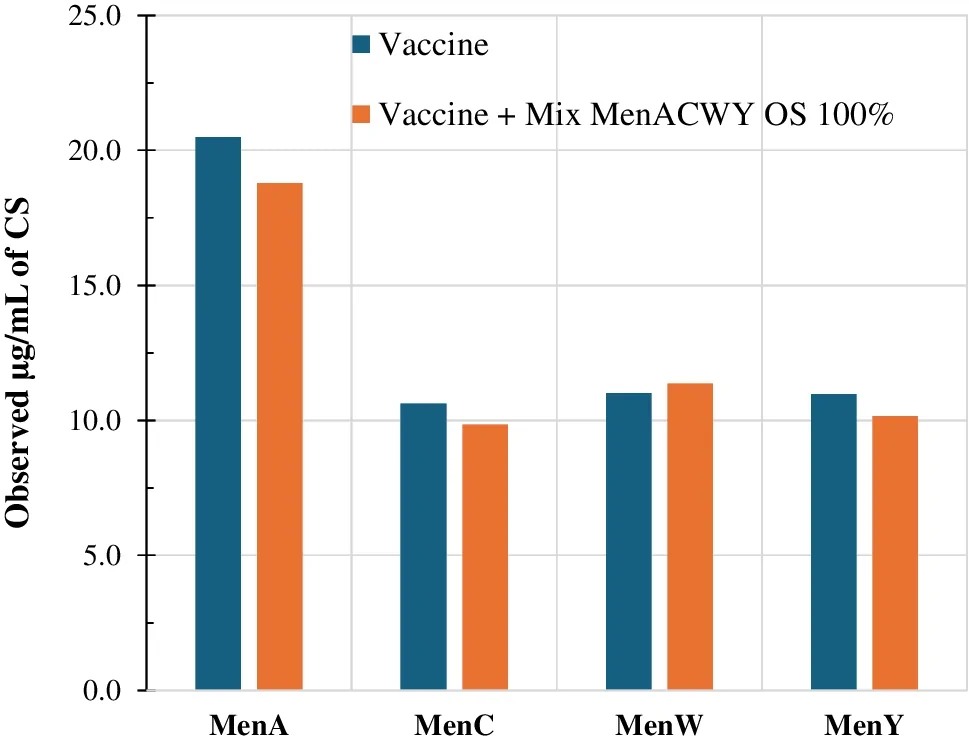
Assessment of no impact by free saccharide in CS quantification analyzing samples reported above. Results are expressed as µg/mL of conjugated saccharide

For an immune-based assay, the specificity of the binding between antibody and ligand is crucial. In this case, the main concern was related to possible cross-reactivity among MenW and MenY conjugates and their beads-mAb complex, due to their strictly similar saccharide repeating units [MenW: → 6) -α-D-Galp-(α1–4)–α-D-Neu5NAc–(α2 →; MenY: → 6) α-D-Glcp-(α1–4)–α-D-Neu5NAc–(α2 →], which differ only at the stereogenic center in C4. Thus, to demonstrate the absence of any cross-reactivity among serotypes, we prepared and analyzed ad hoc formulations, taking out one conjugate at a time, starting from the singular drug substances orthogonally quantified with HPAEC-PAD. All the beads were used in this analysis to verify that the missing conjugated antigen was not detected. The results are expressed as %, of recovery, so the ratio between the µg/mL of conjugated saccharide quantified in each ad hoc formulation and the one in the complete mixture with all the antigens. The results in Table S1 in the Supplementary Material show that recoveries are between 82 and 120%, confirming that there is no cross-reactivity between the conjugates. Consequently, we proceeded to evaluate the specificity against the free saccharides. High free OS levels were evaluated to assess the potential impact on the quantification of conjugated saccharides. This evaluation is critical due to the potential interference arising from saccharide-specific capture facilitated by beads conjugated with monoclonal antibodies specific to serotypes. To address this, we quantified conjugated saccharide in the absence and presence of a spike solution of MenACWY oligosaccharides (OS). Moreover, even the spike solution has been analyzed alone on the same plate as the other two samples. The oligosaccharide mixture was prepared at reduced concentrations, specifically at 10% and 20% of the target concentration (data not shown), as well as at a concentration equivalent to the saccharide titers present in the vaccine (MenA OS: 20.0 µg/mL; MenC OS: 10.0 µg/mL; MenW OS: 10.0 µg/mL; MenY OS: 10.0 µg/mL), which serves to simulate an extreme condition as reported in Fig. 3.

**Fig. 4:**
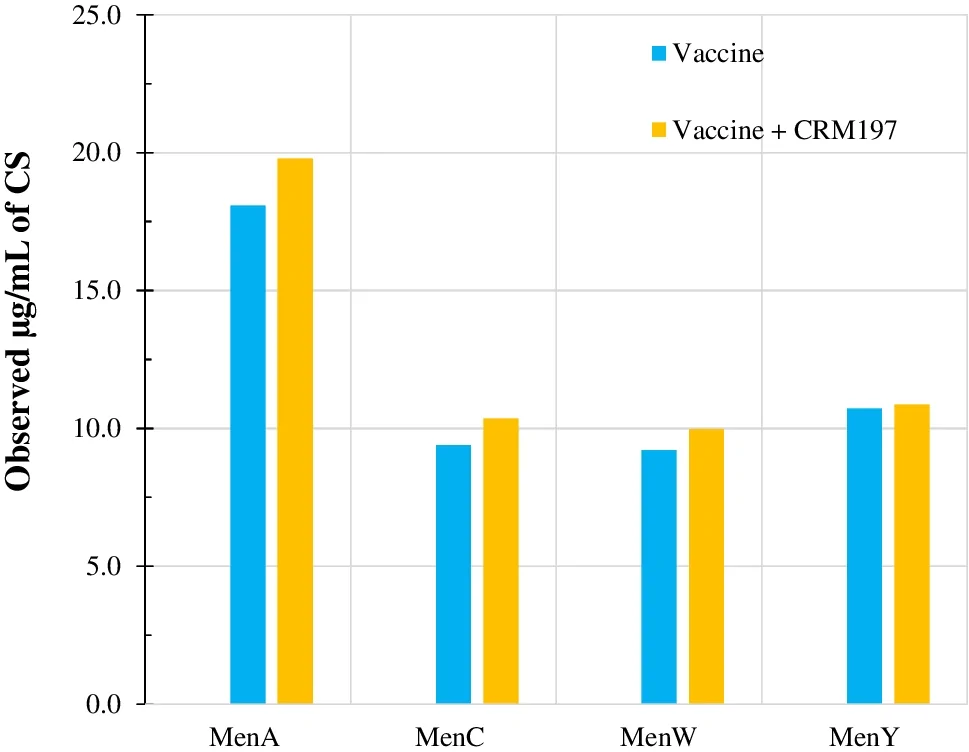
Assessment of no impact by free protein in CS quantification analyzing samples reported above. Results are expressed as µg/mL of conjugated saccharide

In the same way, we investigated a potential impact on antigens quantification by free CRM_197_, which is part of the conjugate detection by anti-CRM polyclonal antibodies. Therefore, we analyzed a vaccine solution at target level, a solution of free CRM_197_ (100 µg/mL), and a vaccine sample at target level mixed with the spike of CRM_197_. The results in Fig. 4 confirm that CRM_197_ does not interfere with conjugated antigens quantification using this assay configuration.

### Method performances

After developing and optimizing the method, we tested the classical analytical performances in a feasibility study conducted at five concentration levels (L1 = 50%; L2 = 75%; L3 = 100%; L4 = 125%; L5 = 150%), which were prepared by diluting the lyophilized vaccine with different volumes to reach the theoretical concentration. These were analyzed and quantified randomly in five analytical sessions. The number of sessions was deemed sufficient to assess preliminary method performances in terms of precision, linearity, and accuracy for each serotype and level. Except for the target concentration (L3), all accuracy levels were analyzed in a single replicate (duplicate wells) in each session. Thus, in the end, this feasibility study was based on five determinations for L1, L2, L4, and L5, and ten determinations for L3.

#### Limit of detection (LOD) and quantification (LOQ)

For conjugated saccharide of each serotype, we reported the lower limit of detection and lower limit of quantification expressed as pg/mL in the well as shown in Table 1. These in-well concentrations of conjugated saccharide correspond to the calculated LOD (LOD = **μ**_MFI (blank)_ + 3**σ**_MFI (blank)_) and LOQ (LLOQ = **μ**_MFI (blank)_ + 10**σ**_MFI (blank)_) starting from the average (µ) and standard deviation (σ) of blank MFI.

**Table 1:** Limit of detection (LOD) and limit of quantification (LOQ)

Serotype	LOD (pg/mL)	LOQ (pg/mL)
MenA	2.0	2.0
MenC	2.6	1.0
MenW	6.3	2.5
MenY	44.3	17.7

#### Precision

The inter-assay precision (intermediate) has been evaluated with one replicate at all five levels across the five independent analytical sessions, as shown in Figure S1 of the supplementary material. Although the limited number of analytical sessions, a final coefficient of variation (CV%) widely under 20% was obtained. As previously cited, for each session, two analytical replicates of L3 (100%) were independently prepared and analyzed, with a resulting intra-assay precision (repeatability) of 9%, 7%, 6%, 6% for MenA, MenC, MenW, and MenY, respectively.

#### Linearity

The method resulted in a good linearity, as reported in Figure S2 of the supplementary material, which shows the regression lines derived by plotting the theoretical (Th.) antigen amount as the dependent variable (Y) and the observed (Obs.) concentrations as the independent variable (X). The latter values were obtained by calculating the average (Avg.) of all sessions. Each serotype presents an R^2^ ≥ 0.99 and a slope between 0.88 and 1.07, indicating a good correlation between the theoretical and observed concentrations investigated from 50 to 150% of the concentration range of each conjugate.

#### Accuracy

**Fig. 5:**
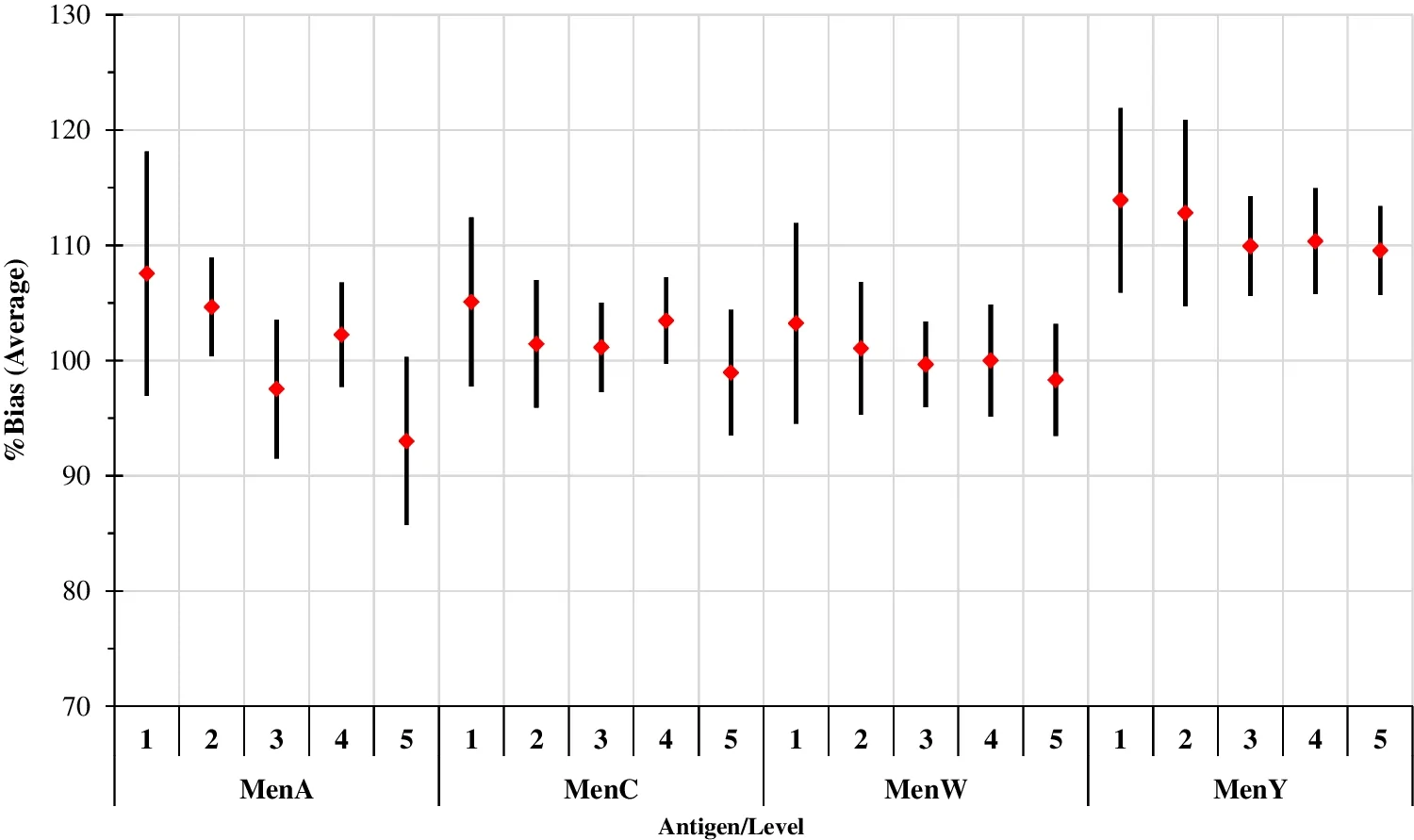
Upper and lower accuracy limits of each serotype and level, calculated with the 90% confidence interval

As illustrated in Fig. 5, the accuracy was reported as an average bias for the five analytical sessions, with the upper and lower limits calculated at 90% confidence intervals. The FDA guidelines recommend a ± 20% of accuracy as the limit for validating immune-based assays [23]. In this context, the method is accurate for all levels and serotypes, except for the lower levels of MenY—L1 and L2—which have slightly larger intervals (122% and 121%). However, this slight overestimation might be dependent on the reference standard titer based on the glucose content, assessed via the orthogonal HPAEC-PAD method.

### Stability monitoring

**Fig. 6:**
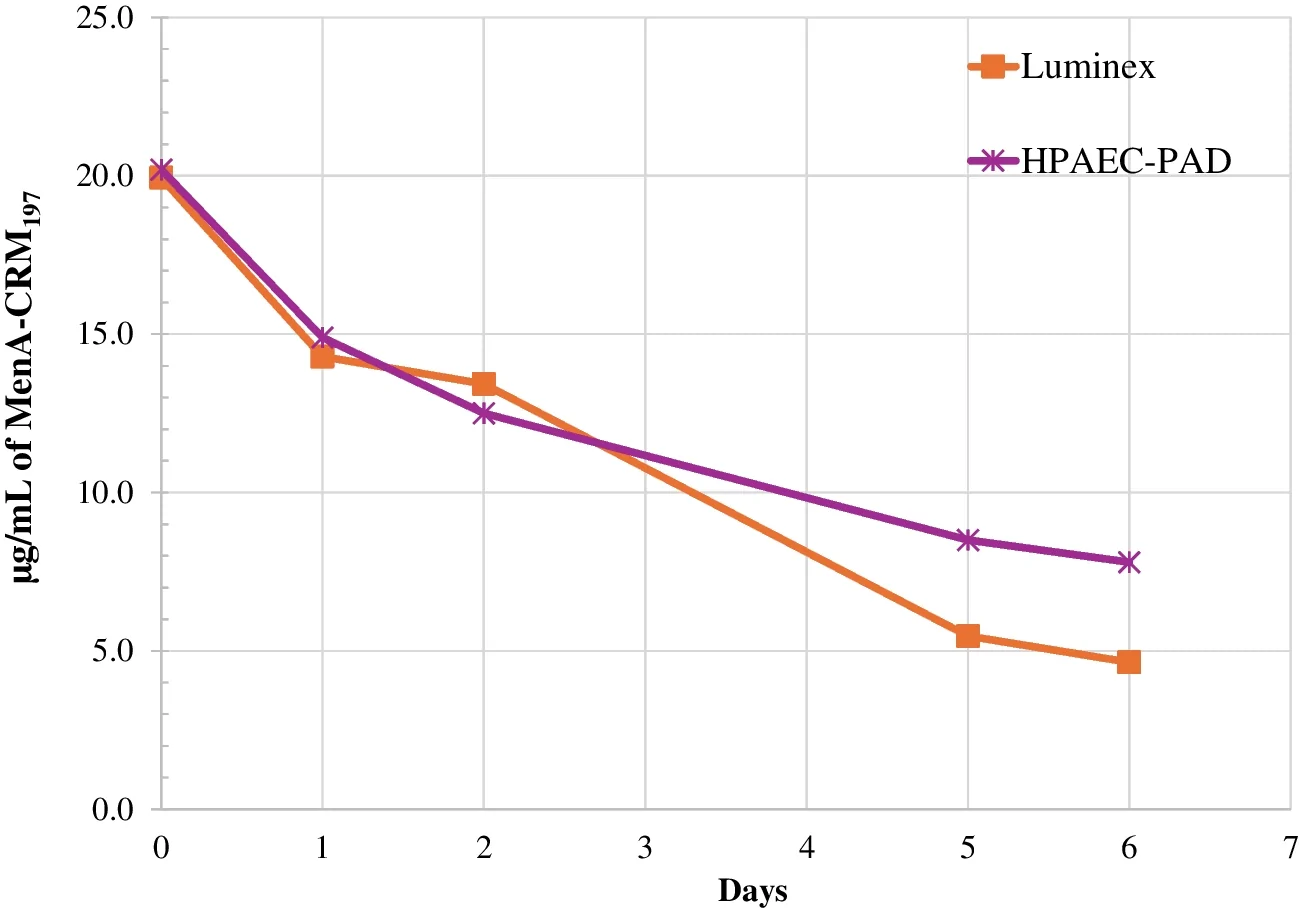
Stability study at 55 °C for 1–2-5–6 days

To evaluate the ability of this multiplex bioassay to monitor the degradation of conjugated saccharides, we employed both chemically induced and long-term stress conditions. Initially, we conducted mild hydrolysis using 1.0 mM trifluoroacetic acid (TFA) at 30 °C for 2 and 4 h, aiming to partially degrade the conjugated saccharides (CS) of the four antigens. Resulting data reported in Figure S3 of the supporting material show a steeper degradation pattern for MenA serotype, containing a phosphodiester bridge in its repeating unit, which makes this conjugated saccharide more susceptible to hydrolysis [24]. On the other hand, the other conjugates are hydrolyzed the same but with a flatter trend with respect to MenA as expected. Consequently, we applied a thermal stress to samples at 55 °C for 1, 2, 5, and 6 days, as done in a previous study on the same vaccine for the validation of an HPAEC-PAD method targeting MenA-CRM_197_ conjugated saccharide [16]. Results graphed in Fig. 6 showed a rapid decrease in CS content. However, a mismatch emerged between Luminex and HPAEC-PAD comparing the results for the more stressful conditions (5 and 6 days).

**Fig. 7:**
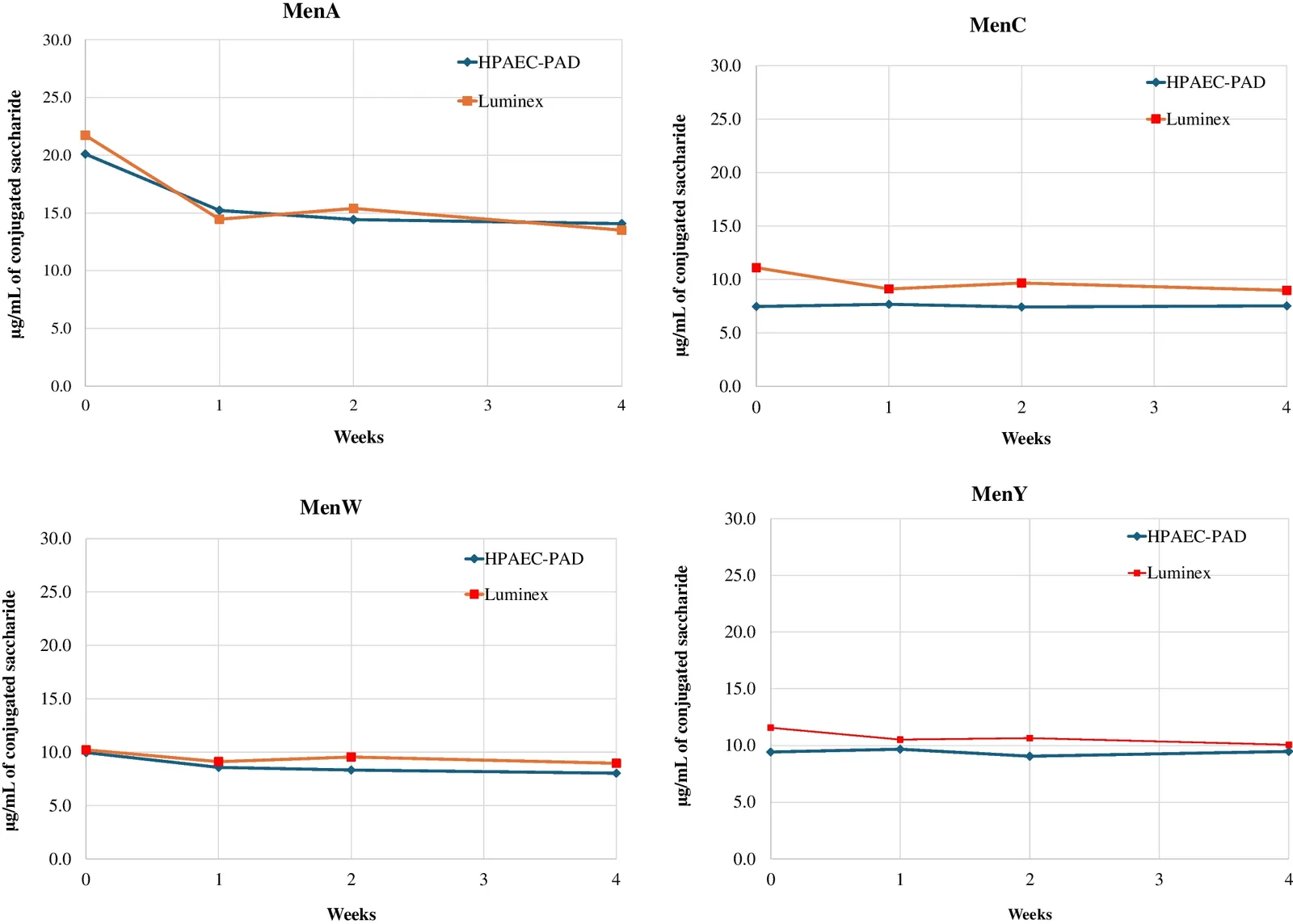
Stability study at 25 °C up to one month of **MenA**, **MenC**, **MenW**, and **MenY** respectively. Results were expressed as conjugated saccharide concentration

As demonstrated by Pecetta S. et al. [25], the transition midpoint temperature (Tm) of CRM_197_ is 46.3 °C, and is even lower in glycoconjugates, depending on the degree of glycosylation, as was studied for MenA-CRM_197_ conjugates [25]. Therefore, a possible reason for this underestimation could be related to the high temperature used, which was above the midpoint temperature of the carrier protein. The consequent misfolding of the carrier protein at this temperature should have interfered in both capture and detection. This suggestion has also been confirmed by fluorescence analysis observing emission spectra at 280 nm aimed at assessing protein folding (Data not shown). Consequently, we carried out a long-term stability study by incubating the reconstituted vaccine sample at 25 °C for 1, 2, and 4 weeks. As shown in Fig. 7 all the time points were analyzed by Luminex and the previously cited validated HPAEC-PAD method targeting CS content [16]. Results confirmed a good alignment between the two methods, further confirming the suitability of the Luminex assay to assess glycoconjugate content as compared to the current standard of care. This is indirectly confirming the evidence of correlation between Conjugate Saccharide quantification vs Total – Free Saccharide quantification previously shown by Rech et al. Additionally, this immunoassay was able to discriminate not only antigen integrity but also that of its carrier.

## Discussion

In this article, we showed the development and the feasibility study of a Luminex analytical immune-assay as an alternative to the classical physico-chemical methods, such as HPAEC-PAD or colorimetric, for the quantification of glycoconjugate vaccines. This bioassay enables multiplexed, high-throughput detection of Menveo conjugates by employing a sandwich configuration, consisting of saccharide-based capture that is specific to each serotype and detection based on the carrier protein. The designed configuration aims to quantify, without any sample pre-treatment, only the conjugated component, which is the only active part of this type of vaccine. After identifying a suitable amount of mAb to be coupled on the beads and the working dilution for both primary and secondary antibodies, we shifted our focus to establish a unified condition for analyzing all serotypes in a single plate. The main criticalities were related to the double concentration present in the vaccine of MenA-CRM_197_ compared to the other three serotypes and the lower affinity of the anti-MenY mAb, which necessitated a larger sample amount. Despite this, we found a unique condition for analyzing all the antigens in one well, enhancing the high throughput of this assay. Before delving deeper, we assessed the effective specificity of this assay, both between the conjugates and against impurities. In this investigation, the immunoassay specifically targeted the conjugated saccharides, showing no cross-reactivity among serotypes, particularly among MenW and MenY, which are closely related having similar saccharide structures. Evaluation of potential interference from free saccharides and free CRM_197_ protein confirmed no impact on quantification, underscoring the specificity of the assay in distinguishing conjugated from free components. Subsequently, a feasibility study conducted across five concentration levels (L1 = 50%; L2 = 75%; L3 = 100%; L4 = 125%; L5 = 150%) demonstrated consistent and reliable quantification, with accuracy maintaining well within the ± 20% range, except in the lower concentration levels for MenY (L1 and L2), possibly due to reference standard determination with orthogonal techniques. Lower limits of detection (LLOD) and quantification (LLOQ) were identified, confirming the assay's sensitivity and capability to detect low concentrations of conjugated saccharides. In the end, three different studies of stability were conducted to assess if this multiplex Luminex assay could be a stability-indicating method (SIM). In the first instance, the assay demonstrated effectiveness in monitoring the stability of conjugated saccharides under mild acid conditions (TFA 0.5 mM at 30 °C for 2 and 4 h). For this reason, we repeated the stability as conducted in a previous study (55 °C for 1, 2, 5, and 6 days) to assess the equivalence between HPAEC-PAD and our Luminex method. However, a discrepancy of results was observed in the last two time points due to the elevated temperature used, which exceeded the transition midpoint temperature (Tm) of CRM_197_, known to be 46.3 °C and even lower in its glycoconjugates. For this reason, we reduced the temperature for the study, incubating at 25 °C for different weeks. Indeed, in this case, the stability time points matched with the same validated and orthogonal physico-chemical method previously compared, demonstrating an equivalency of results for all serotypes.

## Conclusions

With the increasing multivalency of vaccines, traditional physico-chemical methods are facing limitations, pushing a shift towards high-throughput and specific multiplex immune-assays. The study highlights the possible improvement which Luminex technology can offer to the vaccine quality control by overcoming the limitations of traditional methods. The selected configuration has the aim of developing a free pre-treatment and direct method, which could target only the conjugated saccharide. This direct estimation of the active ingredient in the drug product is an already known approach that can be adopted and accepted by regulatory agencies only monitoring the free saccharide along all the production steps and until the final formulation mixing. Applying this method on Menveo, we considered only MenACWY-CRM_197_ conjugates and so on relative IgG for sandwich detection. This could be considered a restriction with respect to the classical physico-chemical methods, which are not impacted by the change of the carrier protein, such as the MenACWY-TT of MenQuadfi or Nimenrix. However, switching the anti-carrier antibody such as anti-TT or anti-DT can be easily adopted without any expected impact on the assay format or performance being the primary detection, and therefore used without manipulation. This remains applicable also in case of multiple carrier proteins within the same DP composition, being the assay configuration specific to the PS and to the carrier. This requires experimental confirmation. In conclusion, this technique can be considered as a potential alternative analytical platform, and this direct approach as a valid solution for streamlining vaccine analysis processes, enhancing high-throughput, and providing robust data for conjugated saccharide. Overall, the results highlight the robust analytical performance of the Luminex-based multiplex immunoassay, providing significant advantages over traditional methods in terms of throughput, simplicity, and efficiency.

## Supplementary Information

Below is the link to the electronic supplementary material.

Supplementary Material 1 (DOCX 652 KB)

## Data Availability

Data are available from the authors by request.
