# Supplementary material for: A multiplex method for high-throughput quantification of conjugated saccharide in glycoconjugate vaccines
Source: Anal Bioanal Chem. 2025 Nov 29;418(18):5871–80. doi: 10.1007/s00216-025-06249-5 (PMC13541847; doi:10.1007/s00216-025-06249-5)
Supplement: Supplementary file 1 [file 216_2025_6249_MOESM1_ESM.docx]

**Supplementary Material**

A multiplex method for high-throughput quantification of conjugated saccharide in glycoconjugate vaccines

Giovanni Santostefano^1^, Alessio Corrado^2^, Carmine Malzone^2^, Leonardo Mori^2^, Diego Amendola^2^, Stefania Berti^2^, Valeria Di Bussolo^1^ and Riccardo De Ricco^2,^*

^1^ University of Pisa, Pharmacy Departement, Via Bonanno Pisano 33, 56126, Pisa

^2^ Technical R&D, GSK , Via Fiorentina 1, 53100, Siena

*Correspondence: [riccardo.x.de-ricco@gsk.com](mailto:riccardo.x.de-ricco@gsk.com)

- **Figure S1** Inter-assay precision among all serotypes for each level based on one replicate for each of the five independent runs.
- **Figure S2** Linearity as a regression line obtained plotting the theoretical concentration (Y) and the average concentration of conjugated saccharide observed along the analytical sessions of the study. Are reported for each serotype its relative equation and R^2^ .
- **Figure S3** Results of a stress study performed using trifluoroacetic acid (0.5 mM) at 30 °C under agitation (1000 rpm) for 2 and 4 hours. The results are expressed as µg/mL of conjugated saccharide for all the four antigens at three time points (0-2-4 h).

- **Table S1.** Recovery percentage of each conjugated saccharide (CS) in every mixture formulated without one conjugate at time. This percentage was given by the ratio between the µg/mL of observed CS in the ad hoc formulate and µg/mL observed in the complete tetravalent formulate, so the MenACWY vaccine itself.

| **Serotype** | **Session** | **MenCWY (no A)** | **MenAWY (no C)** | **MenACY (no W)** | **MenACW (no Y)** |
| --- | --- | --- | --- | --- | --- |
| **MenA** | 1 | 0 | 109 | 100 | 101 |
|  | 2 | 0 | 115 | 114 | 100 |
| **MenC** | 1 | 84 | 0 | 102 | 119 |
|  | 2 | 90 | 0 | 120 | 120 |
| **MenW** | 1 | 82 | 92 | 0 | 118 |
|  | 2 | 91 | 103 | 0 | 114 |
| **MenY** | 1 | 88 | 99 | 110 | 0 |
|  | 2 | 88 | 101 | 125 | 0 |
